# Does social health insurance prevent financial hardship in Mongolia? Inpatient care: A case in point

**DOI:** 10.1371/journal.pone.0248518

**Published:** 2021-03-31

**Authors:** Javkhlanbayar Dorjdagva, Enkhjargal Batbaatar, Mikael Svensson, Bayarsaikhan Dorjsuren, Munkhsaikhan Togtmol, Jussi Kauhanen

**Affiliations:** 1 Institute of Public Health and Clinical Nutrition, Faculty of Health Sciences, University of Eastern Finland, Kuopio, Finland; 2 Department of Social Sciences, Faculty of Social Sciences and Business Studies, University of Eastern Finland, Kuopio, Finland; 3 School of Public Health and Community Medicine, Institute of Medicine, University of Gothenburg, Gothenburg, Sweden; 4 Department of Health Systems Governance and Financing, WHO, Geneva, Switzerland; 5 Ministry of Health, Ulaanbaatar, Mongolia; University of Western Australia, AUSTRALIA

## Abstract

**Background:**

Protecting people from financial hardship and impoverishment due to health care costs is one of the fundamental purposes of the Mongolian health system. However, the inefficient, oversized hospital sector is considered one of the main shortcomings of the system. The aim of this study is to contribute to policy discussions by estimating the extent of catastrophic health expenditure and impoverishment due to inpatient care at secondary-level and tertiary-level public hospitals and private hospitals.

**Methods:**

Data were derived from a nationally representative survey, the Household Socio-Economic Survey 2012, conducted by the National Statistical Office of Mongolia. A total of 12,685 households were involved in the study. “Catastrophic health expenditure” is defined as out-of-pocket payments for inpatient care that exceed a threshold of 40% of households’ non-discretionary expenditure. The “impoverishment” effect of out-of-pocket payments for inpatient care was estimated as the difference between the poverty level before health care payments and the poverty level after these payments.

**Results:**

At the threshold of 40% of capacity to pay, 0.31%, 0.07%, and 0.02% of Mongolian households suffered financially as a result of their member(s) staying in tertiary-level and secondary-level public hospitals and private hospitals respectively. About 0.13% of the total Mongolian population was impoverished owing to out-of-pocket payments for inpatient care at tertiary-level hospitals. Out-of-pocket payments for inpatient care at secondary-level hospitals and private hospitals were responsible for 0.10% and 0.09% respectively of the total population being pushed into poverty.

**Conclusions:**

Although most inpatient care at public hospitals is covered by the social health insurance benefit package, patients who utilized inpatient care at tertiary-level public hospitals were more likely to push their households into financial hardship and poverty than the inpatients at private hospitals. Improving the hospital sector’s efficiency and financial protection for inpatients would be a crucial means of attaining universal health coverage in Mongolia.

## 1. Background

Universal health coverage (UHC) is one of the overarching health objectives of the global Sustainable Development Goals [1]. It is intended as a global means of ensuring equity of access to quality health care and provision of financial protection for everyone [2, 3]. Primary health care (PHC) is recognized as a key strategy to reach this ambitious goal, while the role of the hospital sector (where most health sector resources are consumed) is often overlooked in international and local policy reviews and studies. However, some argue that hospitals are a cornerstone for sustaining UHC [4]. This may be particularly relevant for a country like Mongolia, with its large and inefficient hospital sector [5].

Before 1990, Mongolia’s health system was based on the Semashko model, which had been developed in the Soviet Union. Accordingly, the state was responsible for both providing and financing health services [6]. Documentation shows that, under this system, public health infrastructure improved dramatically and the number of hospitals rose. Structural development brought nationwide access to public health and health care services, despite the country’s relatively large territory and low population density [6]. Thus, the greatest improvement in health outcomes took place during the period when this model was applied [5]. However, the system had many shortcomings, including the large, inefficient hospital sector’s provision of low-quality services and poor PHC development [7].

After 1990, during Mongolia’s transition from central planning to a free market economy, the health sector faced financial constraints like the other sectors. With international assistance, the government successfully implemented health care reforms, including implementation of compulsory social health insurance (SHI), establishment of PHC, development of the private health sector and the national decentralization policy, aimed at ensuring equity in health care and financial protection in the sector [6]. Consequently, 95.6% of the total population has been covered by SHI since 2017 [8], with universal free access to PHC that is fully funded by the state [6]. However, despite these achievements, the Mongolian health system ranked 145th of 191 countries [9], which indicates that the current high SHI and free PHC coverage are insufficient to sustain UHC [10–13]. The inefficient, oversized hospital sector is considered one of the main shortcomings of the system [6]. Since the hospital sector plays a major role in delivering health care in Mongolia, we believe that improving hospital efficiency and enhancing service quality are crucial aspects of sustaining UHC in the country.

Inpatient care is provided by public and private hospitals in Mongolia. There are 13 tertiary-level hospitals (multispecialty central hospitals and specialized centers), 28 secondary-level hospitals (provincial or *aimag* general hospitals and Ulaanbaatar district hospitals), and 5 regional diagnostic and treatment centers [14]. All these higher-level public hospitals are located in the capital and provincial centers. In addition to the large public hospital sector, private hospitals have proliferated since 1990; by 2016, there were 234 [14]. Most of the private hospitals are located in urban areas, in response to the clients’ ability to pay [15]. In rural areas, inpatient care is provided by *soum* (district) health centers that are fully funded by the state.

The number of hospital beds per 1,000 people in Mongolia is 6.8, which is considerably higher than in most countries [16]. Moreover, inpatient care accounts for over half of expenditure on both total health care (55% in 2009) [6] and SHI (53.8% in 2015) [14]. Public hospitals are funded through the state budget (based on the line-item budget method), the SHI fund (based on case-related hospital payments using diagnosis-related groups (DRGs), and user charges [15, 17]. On average, more than half of public hospitals’ revenue comes from the state budget and 30% from the SHI fund, while up to 10% is derived from user charges. For private hospitals, 70–90% of revenue comes from user charges and up to 30% from the SHI fund, on average [15, 17].

The recent article demonstrated that, given weak regulation, the growth of private hospitals creates duplication of health services in both the public and the private health sector [15]. Furthermore, unnecessary hospital admission is common in both public and private hospitals, owing to poor PHC gatekeeping systems, health-seeking behavior, and financial incentives for inpatient care [15].

It is well known that health (care) equity and catastrophic health expenditure (CHE) are the main indicators of UHC and require a sustainable monitoring process. In our previous study, we found that 5.5% of the total households faced CHE and 0.78% of the Mongolians were pushed into poverty by their health care payments in 2012 [13]. But the study provides no evidence on CHE and impoverishment effects in relation to health care types (inpatient and outpatient care), medicine costs, or levels and types of health care provider.

This study therefore goes beyond our previous work. We analyze the extent of CHE on secondary-level and tertiary-level inpatient care, and the impoverishment effects of this care, at public and private hospitals. Since *soum* health centers are primary-level units fully funded by the state, we exclude them from this analysis. We hypothesized that the extent of CHE and impoverishment effects due to inpatient care was smaller for care at public than at private hospitals. This is because when the insured use inpatient care at public hospitals they are required to make co-payments of 10–15% [18]. On the other hand, user charges are the main source of income for private hospitals, although some people choose private hospitals because access to them is easier and waiting time shorter. This applies although there is no evidence of whether utilization of private hospitals causes CHE among users. The aim of the study is to contribute to policy discussions by estimating the extent of CHE and impoverishment due to inpatient care at secondary-level and tertiary-level public hospitals and private hospitals.

## 2. Methods

### 2.1 Data

The present study used the data obtained from a national survey, the Household Socio-Economic Survey 2012 (HSES), conducted by the National Statistical Office of Mongolia (NSO). The aim of the survey is to “evaluate and monitor the income and expenditure of households, update the basket and weights for consumer price index, and offer inputs to the national accounts” [19]. The survey questionnaire includes a health module that provides information on health outcomes, utilization of health services, and health expenditures. It allows us to estimate the incidences of CHE and impoverishment. As we mentioned before in our previous study, we estimated the incidences of CHE and impoverishment, in general, using HSES 2012 [13]. Using the same HSES 2012 in the present study, as a continuation of our previous study, will help policymakers and readers understand the role of the hospital sector in sustaining UHC by comparing extents of CHE due to inpatient care and CHE due to the total health care expenditure.

HSES 2012 covered 12,811 households comprising 47,908 individuals in 2012. Detailed information on survey methodology (aim, sampling methods, data collection, and quality control) is given elsewhere [19]. The main inclusion criterion was for household members to have stayed in different levels or types of hospital during the study period. When this criterion was applied, 12,685 households were retained in the study.

### 2.2 Variables

The third section of HSES 2012 focuses on respondents’ health status, health care utilization, and payments. All the members of each household were asked whether they had been in hospital in the past 12 months and, if so, what kind of hospital it had been. In this paper, “type of hospital” refers to either public or private and “level of hospital” refers to either public secondary level or public tertiary level. From the data set, we produced three dummy variables: (a) a household with at least one member who had stayed in a private hospital only; (b) a household with at least one member who had stayed in a secondary-level hospital only; and (c) a household with at least one member who had stayed in a tertiary-level hospital only. There were 126 households whose members had stayed in hospitals of more than one level or type of hospital during the study period. These households were eliminated from the analysis because of their incompatibility with the aim of the study, which was to measure and compare the extent of catastrophic inpatient health expenditure associated with different hospital types and levels.

A household’s monthly out-of-pocket payments (OOPs) for inpatient care were estimated in terms of the two categories of expenditure. The first was “direct payments,” comprising payments and/or co-payments for hospital care and inpatients’ purchases of medicines during their hospital stay. The second, “non-medical indirect costs,” included costs of transportation, accommodation, meals, and gifts for medical staff during the stay. Although all hospitals provide meals for all their inpatients, it is common throughout the country for patients to receive meals from home, relatives or friends [20].

We generated a categorical variable, “expenditure quintiles,” based on total household expenditure per capita (quintile 1 = poorest 20%, quintile 2 = second-poorest 20%, quintile 3 = middle 20%, quintile 4 = —second-richest 20%, quintile 5 = richest 20%). Location is a binary variable (urban or rural).

### 2.3 Measuring catastrophic health expenditure

First, we estimated the incidence of catastrophic payments due to inpatient care.

In the literature, there are several approaches to defining CHE [21, 22]. In this study, CHE is considered to exist if OOPs for inpatient health care exceed a threshold of 40% of a household’s non-discretionary monthly expenditure [21] which is also known as its “capacity to pay” [23]. A household’s “non-discretionary expenditure” refers to household non-food spending, which is the difference between households’ total expenses and their spending on food.

The incidence (H) of catastrophic payments, which refers to the proportion of households that incurred catastrophic inpatient payments, is estimated as follows [21, 22].

$$H=\frac{1}{N}\sum_{i=1}^{N}E_{i}$$ (1)

where N denotes the sample size. *E* represents an indicator such that *E*_*i*_ = 1 if $\frac{T_{i}}{nf{\left(x\right)}_{i}}>40$, and is otherwise zero. Where T denotes OOPs for inpatient care, nf(x) is capacity to pay or a household’s non-discretionary expenditure per month.

### 2.4 Measuring impoverishment effect of OOPs for inpatient care

We estimated poverty headcount ratios on two occasions (before and after the hospital stay).

First, the gross of the health payments poverty ratio (*HP*^*gross*^) was obtained. This shows the incidence of poverty at national level before inpatient care payments [21, 22]:

$$HP^{gross}=\frac{\sum_{i=1}^{N}s_{i}p_{i}^{gross}}{\sum_{i=1}^{N}s_{i}}$$ (2)

where $P_{i}^{gross}$ is equal to 1 if per capita total expenditure of household *i* is below the poverty line and otherwise 0, *s*_*i*_ is household size, and N denotes the number of households in the sample.

Second, the net health payments poverty ratio can be obtained in a similar way [21, 22]:

$$HP^{net}=\frac{\sum_{i=1}^{N}s_{i}p_{i}^{net}}{\sum_{i=1}^{N}s_{i}}$$ (3)

where $p_{i}^{net}$ is equal to 1 if the per capita total expenditure of the household is below the poverty line, and otherwise 0.

Finally, we estimated the impoverishment effect of OOPs for inpatient care. This is easy to estimate, by subtracting the poverty level with the gross total of OOPs (before health care payments) from the poverty level with the net total of OOPs (after health care payments) or *HP*^*net*^ − *HP*^*gross*^ [21, 22].

This paper applied the Mongolian national poverty line that is estimated by the NSO annually. In 2012, the national poverty line corresponded to a monthly income of MNT 118,668 (the Mongolian tugrik was worth USD 199.64 at purchasing power parity in 2012) [24].

## 3. Results

The distributions of inpatient care utilization, in terms of expenditure quintiles and geographical areas in Mongolia, are represented in Table 1 by the chi-square test. In the reference year, 9.21%, 11.18%, and 3.97% of all households reported that at least one member of the household used inpatient care at a tertiary-level, a secondary-level public hospital, and a private hospital, respectively. Results showed that urban households used inpatient care significantly more than rural households, at all types and levels of hospital. Rich households reported more inpatient care utilization (p<0.01) than poor ones, except at secondary-level hospitals.

**Table 1:** Distributions of inpatient care utilization by expenditure quintiles and location in Mongolia, 2012 (%).

Inpatient care utilization	Expenditure quintile (%)**					Location (%)		All (N = 12,685)
	1	2	3	4	5	Urban	Rural	
Private hospital	1.75	2.76	3.49	4.91	7.19*	5.12	2.58*	3.97
Secondary-level hospital	10.44	11.69	11.46	11.16	11.15	13.31	8.59	11.18
Tertiary-level hospital	5.77	7.34	9.61	11.20	12.40*	10.96	7.07*	9.21

* Statistically significant difference (p < 0.01)

** Expenditure quintile (1 = poorest 20 percent, 2 = second poorest 20 percent, 3 = middle 20 percent, 4 = second richest 20 percent, 5 = richest 20 percent)

### 3.1 OOPs for inpatient care at different hospitals

**Fig 1:**
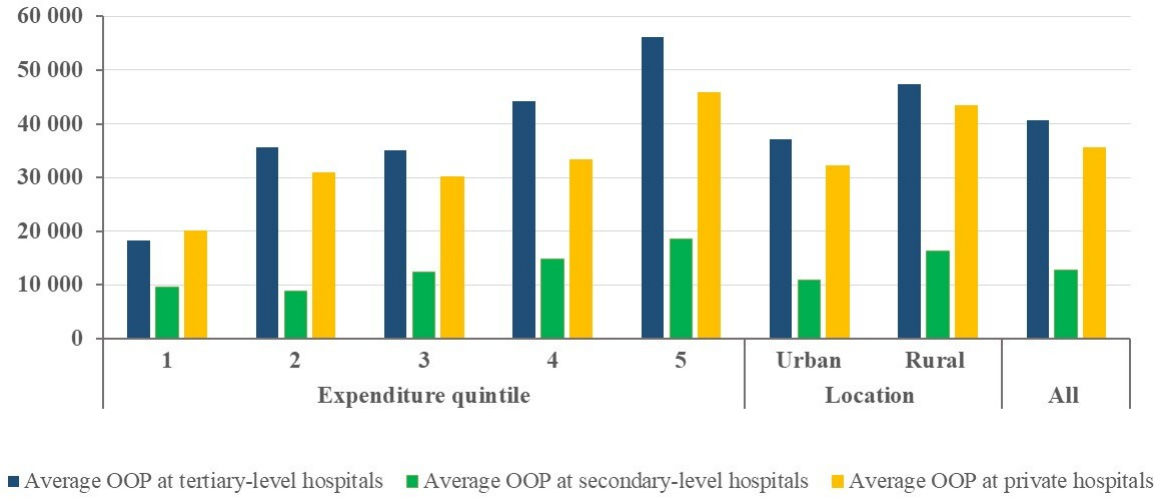
Out-of-pocket payments (MNT) for inpatient care at different hospitals by expenditure quintiles and locations.

Fig 1 illustrates average OOPs for inpatient care by type and level of hospital. The results show that inpatients at tertiary-level hospitals reported the highest OOPs (MNT 40,600.8), followed by those at private hospitals (MNT 35,499.3) and secondary-level hospitals (MNT 12,762.9).

Average OOPs for inpatient care at tertiary-level and secondary-level public hospitals and private hospitals for households in rural areas (MNT 47,285.3, 16,224.0, and 43,364.9 respectively) were higher than for those in urban areas (MNT 37,051.5, 10,924.1, and 32,229.3 respectively).

Additionally, the household expenditure quintile rises as average OOPs for inpatient care increase, irrespective of hospital type and care level.

**Fig 2:**
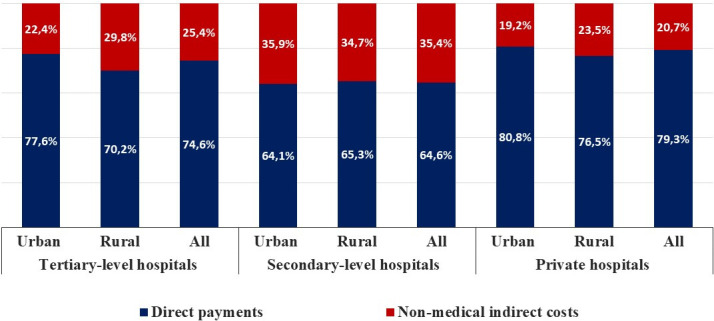
Structures of average out-of-pocket payments for inpatient care by different levels and types of hospitals in Mongolia.

Structures of average OOPs for inpatient care by different levels and types of hospital in Mongolia are shown in Fig 2: direct payments account for between 64.1 and 80.8% of OOPs across all hospitals, while non-medical indirect costs as proportions of OOPs at different hospitals, range from 19.2 to 35.9%.

### 3.2 Catastrophic health expenditure

Table 2 shows the extent of CHE due to hospital stays at tertiary and secondary-level public hospitals and private hospitals. Results show that 0.31%, 0.07%, and 0.02% of Mongolian households suffered financially because of one or more of their members staying at tertiary-level hospitals, secondary-level hospitals, and private hospitals, (Table 2, columns 2, 4. and 6). Regarding tertiary-level hospitals, urban households had higher rates of CHE than rural ones, while the opposite was true of secondary-level hospitals. For private hospitals, there was no difference in the rates of CHE found in rural and urban locations. We also estimated the rates of CHE among households with at least one member staying in hospital (Table 2, columns 3, 5, and 7).

**Table 2:** Extent of catastrophic health expenditure on inpatient care, by hospital type, in Mongolia.

	Tertiary-level hospitals		Secondary-level hospitals		Private hospitals	
	All households	Households with member(s) using inpatient care	All households	Households with member(s) using inpatient care	All households	Households with member(s) using inpatient care
Location						
Urban	0.37%	2.99%	0.03%	0.24%	0.02%	0.39%
Rural	0.19%	3.13%	0.16%	1.89%	0.02%	0.97%
All	0.31%	3.02%	0.07%	0.71%	0.02%	0.50%

Households with members who stayed at tertiary-level hospitals had the highest rates of CHE (3.02%): roughly four to six times higher than those with members staying at secondary-level (0.71%) and private hospitals (0.50%). In rural areas, tertiary-level hospital users had the highest rate of CHE (3.13%), followed by secondary-level hospital inpatients (1.89%) and private-hospital users (0.97%).

In urban areas, stays in tertiary-level hospitals caused the highest rate of CHE (2.99%). Households using inpatient care at secondary hospitals and private hospitals incurred financial hardship relatively infrequently (0.24% and 0.39% respectively).

### 3.3 Impoverishment due to inpatient care, by hospital type

Table 3 shows the absolute and relative increases in poverty headcount ratios due to OOPs for inpatient care at different types of hospital. The poverty headcount ratio was estimated as 22.26% in 2012, using household total expenditure as a proxy for living standards. The results reveal that 0.13% of the total Mongolian population was impoverished as a result of OOPs for inpatient care at tertiary-level hospitals.

**Table 3:** Absolute and relative increases in poverty headcount ratios due to out-of-pocket payments.

	Tertiary-level hospitals		Secondary-level hospitals		Private hospitals	
	Absolute	Relative	Absolute	Relative	Absolute	Relative
National level	0.13%	0.59%	0.10%	0.46%	0.09%	0.42%
Urban locations	0.14%	0.74%	0.10%	0.54%	0.14%	0.75%
Rural locations	0.12%	0.40%	0.11%	0.37%	0.00%	0.00%

The OOPs for inpatient care at secondary-level hospitals and private hospitals accounted, respectively, for 0.10% and 0.09% of the total population becoming impoverished in 2012.

Impoverishment effects of OOPs for inpatient care at tertiary-level hospitals and private hospitals were greater in urban areas than in rural, while the opposite was observed at secondary-level hospitals.

## 4. Discussion

There is a need for consistent measurements of OOPs and monitoring of their effects to ensure that financial protection is a driver of UHC. The present study expands on and complements the previous work in several ways [13, 25]. This paper is the first in Mongolia to investigate the extent of catastrophic health expenditure and impoverishment due to inpatient care at secondary-level and tertiary-level public hospitals and private hospitals.

This paper reveals the following key results in relation to health-financing policy in Mongolia. First, our results indicate that patients who utilized inpatient care at tertiary-level public hospitals were relatively more likely to push their own households into financial hardship and poverty. This is certainly an undesirable and unacceptable outcome for the country, which has made many attempts to sustain UHC over the past three decades. One of the main initiatives was the introduction in 1994 of compulsory SHI, under Mongolian law, in citizens’ health insurance [26]. By law, major inpatient services at secondary and tertiary public hospitals must now be included in the SHI benefit package, which is designed to reduce financial barriers to inpatient care [18]. This reform applies to a small number of private hospitals, making their funding conditional on the requirement of accreditation, although private hospitals receive only half as much SHI funding as public hospitals [27]. Accordingly, the main income source of the majority of private hospitals is direct user charges [17]. Furthermore, international evidence shows that private hospitals target higher income groups, which consequently experience financial hardship. After Thailand launched UHC in 2001, for example, the extent of CHE shrank but households utilizing inpatient care at private hospitals were more likely to suffer catastrophic effects from this health expenditure [28]. For this reason, our hypothesis for the present research was that users of inpatient care at public hospitals incur a smaller financial burden than inpatients at private hospitals. However, our results did not support this hypothesis. In Mongolia, there is currently no evidence regarding determinants of patients’ health-seeking behavior and impacts of health-financing methods on health providers’ behavior in Mongolia.

Second, direct payments account for a large share (64.1–80.8%) of OOPs for inpatient care, irrespective of hospital type and level. This can easily be explained for private hospitals, whose main income source is user charges. In public hospitals, the high proportion of direct payments is due to user co-payments and costs of medicines purchased by inpatients during their stay. Inpatients are charged 10% in co-payments at secondary-level hospitals and 15% at tertiary-level hospitals, while SHI covers the remaining costs [18]. This is despite the fact that, in practice, insured inpatients purchase some medicines during their hospital stay if necessary—that is, when the medicines are not available at the hospital [20]. In this case, as a regulation, the costs of medicines purchased should be reimbursed from the SHI fund [18]. Because of their inadequate knowledge of the bureaucratic reimbursement process, inpatients are often unable to obtain reimbursement [29], which may lead to financial hardship. Moreover, some public hospitals offer private rooms that contain only one or two beds and are more comfortable than the standard four- to eight-bed wards. The private rooms are indeed expensive and very few people, even those with high incomes, can afford them. We also find that inpatients at tertiary-level hospitals reported the highest OOPs. It could be explained by the fact that private practice receives only low-risk patients who stay for a short period with no unexpected additional health expenditure, while public practice tends to treat high-risk patients who stay for longer at hospital and face more unexpected OOPs.

Third, our study reveals that, at tertiary-level public hospitals and private hospitals alike, rich people use inpatient care more than the poor. Similar results have been reported in other studies [12]. Several studies documented that at primary health care level, basic health services are not sufficiently available [30] and the referral system is very weak. Owing to the weak referral system, it is common for high-income earners in Mongolia to bypass PHC services [31] and opt for inpatient care at a higher-level hospital. It cannot be fully explained by a combination of a lack of availability of basic health services at primary health care and within specific facilities. For instance, some people are admitted to inpatient care for the purpose of rest and convalescence, using SHI. This may be an example of moral hazard, in that healthy people may exploit this option, although it is intended to be controlled by a cost-sharing method known as “co-payment” [32].

Compared with public hospitals, access to inpatient care in private hospitals is easy and their accommodation is more comfortable. These characteristics induce high-income earners to seek inpatient care there, despite the high costs. Tsevelvaanchig et al. [15] found that inappropriate hospital admission to Mongolian private hospitals’ departments of internal medicine and neurology amounted to 27.66% and 26.2% respectively. Significantly lower proportions of inappropriate hospital admissions (21.74% and 17.14% respectively) were found in the same departments in public hospitals. Furthermore, 19.4% of hospital admissions to departments of traditional medicine in both public and private hospitals were categorized as unnecessary. A supplier-induced demand may exist in private hospitals and affects high-income earners to choose expensive and unnecessary care. It is worth mentioning that, as our previous paper revealed, the rich are more likely to suffer from CHE [13].

The present study shows that rural inhabitants are comparatively unlikely to receive inpatient care at higher-level hospitals, although their health needs are relatively urgent. A possible explanation for this is that inpatient care at *soum* health centers is free of charge for residents of the catchment areas concerned. Moreover, most hospitals are located in urban areas, entailing both financial and non-financial barriers to the rural population’s inpatient care access. Our study shows clearly that rural inhabitants pay higher OOPs during their hospital stay, irrespective of hospital type and level. Furthermore, some livestock herders may avoid using health care services in urban areas, unless their ill-health is severe, because they cannot leave their animals unattended.

According to an Asian Development Bank report [7], there is a risk of the Mongolian health system becoming dual, with the rich using health services at private hospitals and the poor getting their care at public hospitals. However, the results of our series of studies show that, in Mongolia, PHC is concentrated among the poor and health care at the higher-level hospitals among the rich. However, we found no statistically significant differences among expenditure quintiles in inpatient care utilization at secondary-level public hospitals. This may show a relatively low degree of inequality in secondary-level inpatient care, but it certainly requires further analysis.

The study results have the following policy implications in Mongolia. First, PHC is nonetheless central to UHC. However, despite SHI, people suffer financially during their hospitalization at tertiary-level public hospitals, secondary-level public hospitals and private hospitals. This indicates that restructuring the current hospital sector and improving hospitals’ efficiency are also essential to achieving the goal of UHC in Mongolia.

Second, according to the legal rules on SHI, the insured are not intended to buy medicines while staying at public hospitals. This is a common practice in Mongolia and needs to be eliminated. Additionally, although co-payment is a known effective tool for monitoring both users’ and providers’ behavior in the health sector, the threshold of co-payments needs to be reevaluated. The current co-payment thresholds (10% and 15%) may not work well enough in reducing unnecessary hospital admissions among the more affluent. At the same time, they may impose financial hardship on those who are worse off, or prompt them to avoid using inpatient care when they need it. To provide more equitable inpatient care, the implications of co-payment thresholds that vary in line with households’ socioeconomic characteristics need to be explored. Reducing direct payments, including costs of medicines and co-payment, may be an effective means of improving financial protection for inpatients and boosting accessibility of inpatient care for those, particularly the poor and rural inhabitants, whose health needs are relatively acute. To prevent households from incurring financial hardship due to unnecessary health services at private hospitals, one policy concern is to improve the gatekeeping system at PHC and the monitoring system for price and quality of health services at private hospitals.

This study has some limitations. We used the cross-sectional household survey method, which did not allow us to carry out a causal analysis and identify the trend of CHE. In addition, the study found that this expenditure is caused mainly by direct costs, but the data available were insufficient for us to investigate the structure of direct payments in public hospitals. This calls for further research in the area. Additionally, owing to insufficient data, the authors were unable to distinguish the level of care (secondary or tertiary) provided by the private hospitals. Furthermore, the existing data did not allow us to distinguish a privatized secondary-level hospital (one secondary-level hospital was fully privatized following the decentralization reform of the Mongolian health sector) from the public secondary-level hospitals. In our analysis, we eliminated 126 households with one or more members who had stayed at different types of hospital. This was because our aim was to obtain a clear picture of which hospitals’ inpatient care caused the high rates of CHE and impoverishment in Mongolia in 2012. These CHE and impoverishment rates might have been different if we had included the 126 households that were eliminated from the study.

## 5. Conclusion

Although most inpatient care at public hospitals is covered by SHI’s benefit package, households whose members are inpatients at tertiary-level public hospitals are more likely to suffer from CHE than those with inpatients at private hospitals. Improving the hospital sector’s efficiency and financial protection for inpatients may be a crucial means of attaining UHC in Mongolia.

## Abbreviations

CHE: Catastrophic health expenditure
HSES: Household Socio-Economic Survey
MNT: Mongolian tugrik
NSO: National Statistical Office of Mongolia
OOPs: Out-of-pocket payments
PHC: Primary health care
SHI: Social health insurance
UHC: Universal health coverage
